# Marine sourced tripeptide SRP and its sustained-release formulation SRP-PLGA-MS exhibiting antihypertensive effect in spontaneously hypertensive rats and HUVECs

**DOI:** 10.3389/fnut.2024.1423098

**Published:** 2024-06-12

**Authors:** Miaoen Huang, Tianji Wang, Yinghao Wang, Qingyan Deng, Jinjun Chen, Li Li, Hui Luo, Yingnian Lu

**Affiliations:** ^1^Guangdong Key Laboratory for Research and Development of Natural Drugs, Guangdong Medical University, Zhanjiang, China; ^2^College of Pharmacy, Guangdong Medical University, Zhanjiang, China; ^3^Zhanjiang Marine Biomedical Research Institute, Guangdong Medical University, Zhanjiang, China

**Keywords:** *Sipunculus nudus*, SRP-PLGA-MS, ACE, ROS, HUVECs

## Abstract

Biopeptides from *Sipunculus nudus* were reported with good ACE inhibitory activity, and the tripeptide SRP was one with the highest ACE inhibition rate. However, the disadvantage of short half-life limited the development of peptide drugs. Moreover, the distinct mechanism of the peptide inhibiting ACE remained unknown. Thus, in this study, a sustained release formulation of SRP-PLGA-MS was designed and prepared. Its long-lasting antihypertensive effect as well as improvement of vascular pathomorphology was verified in spontaneously hypertensive rat (SHR). In addition, the anti-oxidant activity of SRP in human umbilical vein endothelial cells (HUVECs) was evaluated. The results showed that SRP inhibited the production of ROS and NO, which involve the NADPH oxidase, and Keap1/Nrf2 signaling pathway. This study demonstrated that SRP-PLGA-MS had the potential to develop sustained-release drugs for hypertension treatment.

## 1 Introduction

Hypertension can cause serious cardiovascular disease and irreversible damage to organs (1, 2), and its etiology is complex and involves multiple systems. In particular, vascular endothelial dysfunction is considered a direct factor of hypertension (3). Endothelial dysfunction is caused by the excessive expression or production of extracellular stimulating factors such as AngII, ROS, RNS, ONOO^−^ and other pro-inflammatory cytokines. Therefore, oxidative stress may be a potential factor. Oxidative stress is stimulated by the excessive production of ROS, which impairs vascular function, cardiovascular remodeling, and other damages in hypertensive patients (4, 5).

AngII is one of the strongest vasoconstrictor (3). In the RAS, ACE converts inactive AngI to active AngII (6). Therefore, ACE is a key regulator of blood pressure, and ACE inhibitors are generally considered a strategy for designing antihypertensive drugs. Currently, synthetic ACE inhibitors, including captopril, benazepril, and enalapril, are used as antihypertensive agents. However, these agents can also cause serious side effects such as dry cough, edema and even serious kidney damage (7). Thus, safer and more effective natural products with ACE-inhibitory and antioxidant properties may improve the treatment of hypertension.

SRP, a tripeptide with the amino acid sequence Ser-Arg-Prp, was hydrolyzed and extracted from the sandworm *Sipunculus nudus* L. In our previous study (8), SRP was found to be an ACE inhibitor with an inhibition rate of 88.56% and an IC_50_ value of 0.046 mmol/L. Molecular docking experiments showed that SRP inhibited ACE activity by forming hydrogen bonds with the ACE active site. The structure of SRP is similar to Captopril, a commercial antihypertensive agent. Therefore, SRP is considered a potential antihypertensive agent.

Recently, ACE inhibitory peptides have been extracted from natural seafood, such as sea cucumbers and marine microalgae (9–11). However, all these inhibitory peptides have the disadvantage of short half-lives. After administration, the drug concentration in the blood decreases quickly, leading to large fluctuations in the blood concentration. A new drug delivery system (DDS) has been developed to solve this problem. This system allowed for slow sustained release or maintained a constant release rate of the drug. Microspheres made of polylactic acid co-glycolic acid (PLGA) were the most common DDS. PLGA is biodegradable and biocompatible. The products of PLGA degradation in the body are non-toxic to humans and do not require the secondary elimination of residues (12, 13). In our previous study, DSPE-PEG-SRP formulation was attempted; however, the product purity was not satisfactory (14).

**Figure 1:**
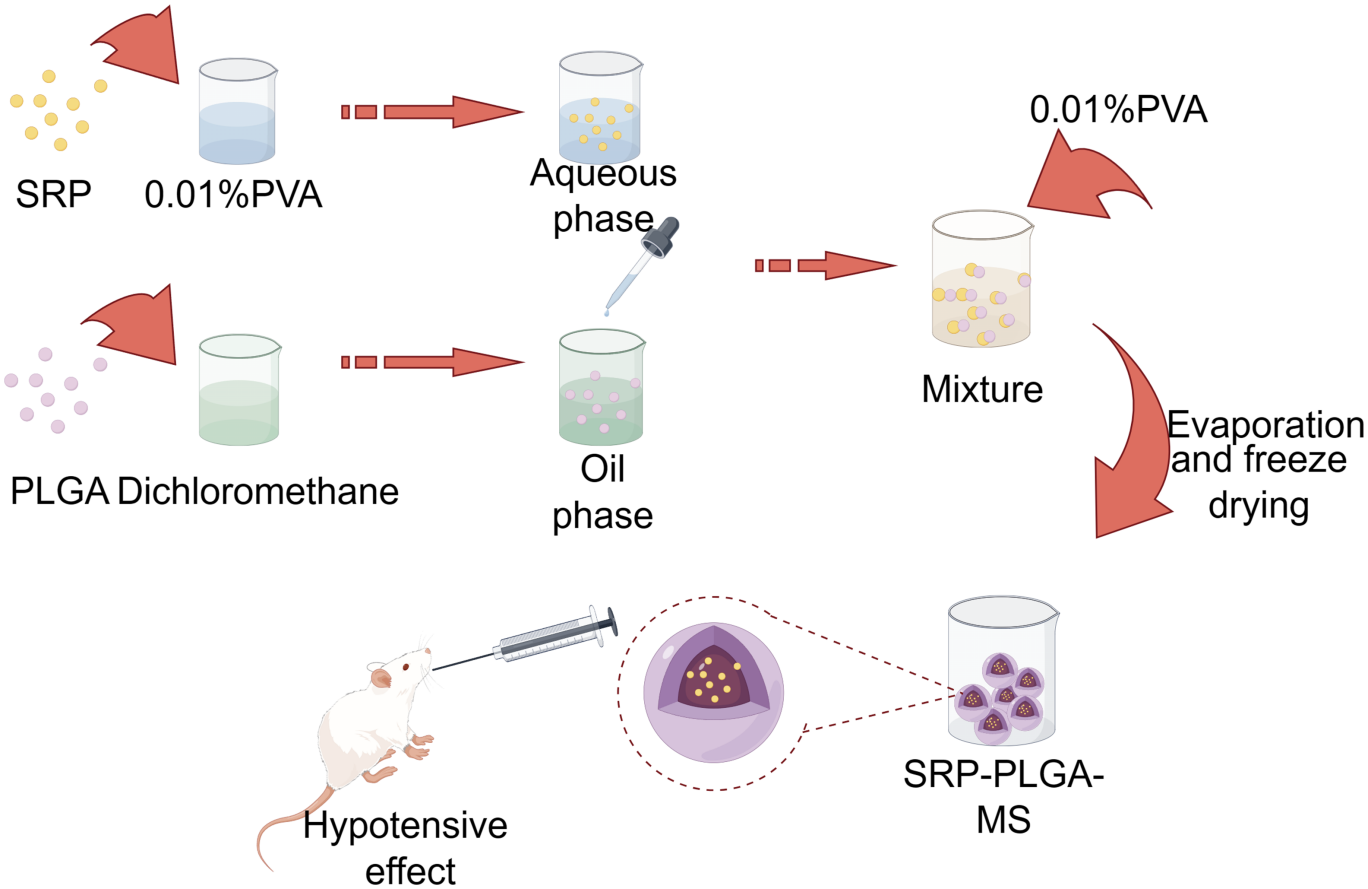
Scheme. Created by Figdraw.com.

In the present study, we designed and prepared SRP-PLGA-MS (Figure 1) and compared its sustained-release effects *in vitro* and *in vivo* to evaluate its antihypertensive effects in SHR. The antioxidant related molecular mechanism of SRP has been explored in HUVECs.

## 2 Materials and methods

### 2.1 Materials

All the chemicals and reagents used were of analytical grade. AngiotensinII (AngII) was purchased from Sigma-Aldrich Chemical Co. DA-FM DA and diphenyltetrazolium (MTT) were purchased from Beyotime Biotechnology. DCFH-DA and PLGA (lactide:glycolide = 50:50, 5 kDa) were purchased from MCE. Primary human umbilical vein endothelial cells (HUVECs; CP-H082) and an endothelial growth medium (CM-H082) were obtained from Procell Life Sciences & Technology Co. Inducible nitric oxide synthase (iNOS, #33424), Nrf2(#41731), Keap1(#41626), GADPH (#52902), NADPH oxidases 4 (#48244), ACE (#49627), Goat Anti-Rabbit lgG (H&L) HRP (#L35009-1) were products of SAB.

### 2.2 Animals

Ten-week-old male SHR (*n* = 16) and SD rats (*n* = 4) were purchased from Guangzhou RAGE Biotechnology Co. (no. 110324231104930174 and no. 44827200007727, respectively). The study strictly adhered to the requirements of animal ethics and welfare (Animal Ethics Certificate No. 20230915-001).

### 2.3 Preparation of SRP-PLGA-MS

SRP-PLGA-MS was prepared using a reported double emulsion method, and the formulation parameters were optimized using two vital indicators of encapsulation efficiency (EE)and drug loading (DL) (15–18). Briefly, PLGA was dissolved in dichloromethane to form the oil phase. Under ultrasonic conditions, an SRP aqueous solution (5 mg of SRP in 0.01% PVA) was added to the oil phase and emulsified to obtain a primary w/o emulsion. The aqueous solution (0.01%PVA) was added dropwise to this primary emulsion with stirring to form a w/o/w double emulsion. The mixture was stirred for 3 h to eliminate dichloromethane and then centrifuged to collect the solid microspheres. After centrifugation, the supernatant was used as the uncoated SRP.

The concentration of uncoated SRP was detected by HPLC, as our previous literature (14), and EE% and DL% were calculated according to the following equations (19, 20):


$$EE\;\left(\%\right)=1-\left(\begin{array}{l} \mathrm{weight of uncoated}\;SRP/\\ \mathrm{total weight of}\;SRP \end{array}\right)\times 100$$

$$DL\;\left(\%\right)=\left(\begin{array}{l} \mathrm{weight of encapsulated}\;SRP/\\ \mathrm{total weight of microspheres} \end{array}\right)\times 100$$

Freshly prepared SRP-PLGA-MS was washed 3 times with distilled water and used for analysis and characterization. The appearance, average particle size, and zeta potential were measured using scanning electron microscopy SEM (TESCAN MIRA LMS, Czech Republic) and a PSS particle size meter (PSS Particle Sizer Inc., United States).

### 2.4 *In vitro* drug release

The *in vitro* release of SRP-PLGA-MS was measured as follows: SRP-PLGA-MS (50 mg) and SRP were suspended in 2 mL deionized water, respectively. The two suspensions were stirred at 100 rpm at 37°C. At designated time, 1 mL of release medium was collected and replaced with 1 mL fresh medium. The amount of SRP released was measured using HPLC (14, 21). Cumulative amount of drug released.


$$E\left(\%\right)=\left(W\;\mathrm{release}/W\;\mathrm{total}\right)\times 100\%$$

### 2.5 *In vivo* study

#### 2.5.1 Measurement of blood pressure

In the SHR with a systolic blood pressure (SBP) above 170 mmHg, each group contained four 10-week-old male rats weighing 200–240 g. SHRs were raised on a standard laboratory diet and maintained at room temperature (24°C). Captopril, SRP (30 mg /kg body weight) and SRP-PLGA-MS (10 mg SRP/kg body weight) were dissolved in saline and orally injected into SHRs. The same volume of saline was orally administered to the SD rats. After oral administration, SBP was measured using a fully automated non-invasive blood pressure measurement system (BP-300A, Chengdu Taimeng Software Co., China) at designated times (22).

#### 2.5.2 Hematoxylin and eosin staining

Thoracic aortic segments from each rat were fixed in 4% paraformaldehyde. They were then embedded in paraffin. These tissues were cut into sections–3-4 μm in size, and stained with hematoxylin and eosin. The media thickness (distance from the inner lamina to the outer lamina) and inner diameter (12 to 6, 3 to 9 o’clock positions) of the thoracic aorta were measured (11, 23). The data were analyzed using Image-Pro Plus 6.0.

### 2.6 Immunohistochemical staining

IHC was performed according to the manufacturer’s instructions. After deaffinity and rehydration, the paraformaldehyde-fixed paraffin-embedded tissue sections were washed twice with PBS and blocked with hydrogen peroxide for 10 min. Antigen extraction was performed. After three washes, sections were blocked for 10 min and incubated with ACE and NADPH oxidase 4 primary antibodies overnight at 4°C. After three washes, the cells were incubated with the secondary antibodies. DAB staining was used to observe the signals and hematoxylin counterstaining was used to observe the nuclei. Strong positive staining was observed under the microscope (23, 24).

### 2.7 Cell culture and cytotoxicity assay of SRP

HUVECs were purchased from Procell (China) and incubated in an endothelial growth medium (CM-H082, Procell) containing 5% FBS and 1% penicillin/streptomycin. The cells were grown at 37°C under 5% CO_2._ HUVECs were seeded at a density of 1 × 10^4^ cells/well in 96-well plates and treated with SRP (312.5, 625, 1,250, and 2,500 μM) for 24 h. Each well was treated with 30 μL MTT for 4 h. The absorbance was measured at 570 nm using a microplate reader (25).

### 2.8 Measurement of intracellular ROS and NO

HUVECs were treated with SRP (50,100 and 200 μM)and Ang II (20 μM) for 24 h. After washing, DCFH-DA (10 μM), Hoechst (10 μM), and DA-FM DA (10 μM) were added and the cells were incubated for 30 min. After 3 times washed, the fluorescent signal (NB-X800LE) was observed and the images were analyzed using ImageJ software (22).

### 2.9 Western blot analysis

Cellular proteins of HUVECs were lysed on ice for 30 min by adding cell lysates containing the enzyme inhibitors. Protein quantification was performed using a bicinchoninic acid (BCA) kit. The quantified samples were then transferred to an electrophoresis gel. Proteins were then transferred onto the NC membranes. The membranes were incubated with 5% defatted milk for 2 h, washed, and incubated with antibodies (iNOS, Nrf2, Keap1, and GADPH) at 4°C overnight. After binding to the secondary antibodies, imaging results were obtained using automated chemiluminescence image analysis.

### 2.10 Statistical analysis

Data are presented as mean ± SD. The One-way analysis of variance (ANOVA) was used for statistical analysis, *p* < 0.05.

## 3 Results

### 3.1 Microsphere preparation and characterization

Different mass ratios of SRP and PLGA were used for the preparation of SRP-PLGA-MS. The EE and DL values are listed in Table 1. The optimal formulation with a mass ratio of 1:5 (SRP/PLGA, w/w) exhibited the highest encapsulation rate (47% ± 8%) and drug load (16% ± 2%).

**Table 1:** Effect of mass ratio of SRP to PLGA on microsphere encapsulation rate and drug loading capacity (Mean ± SD, *n* = 3).

SRP: PLGA	1:4	1:5	1:8	1:10
EE (%)	31 ± 6	47 ± 8	47 ± 3	40 ± 6
DL (%)	16 ± 3	16 ± 2	13 ± 1	6 ± 1

**Figure 2:**
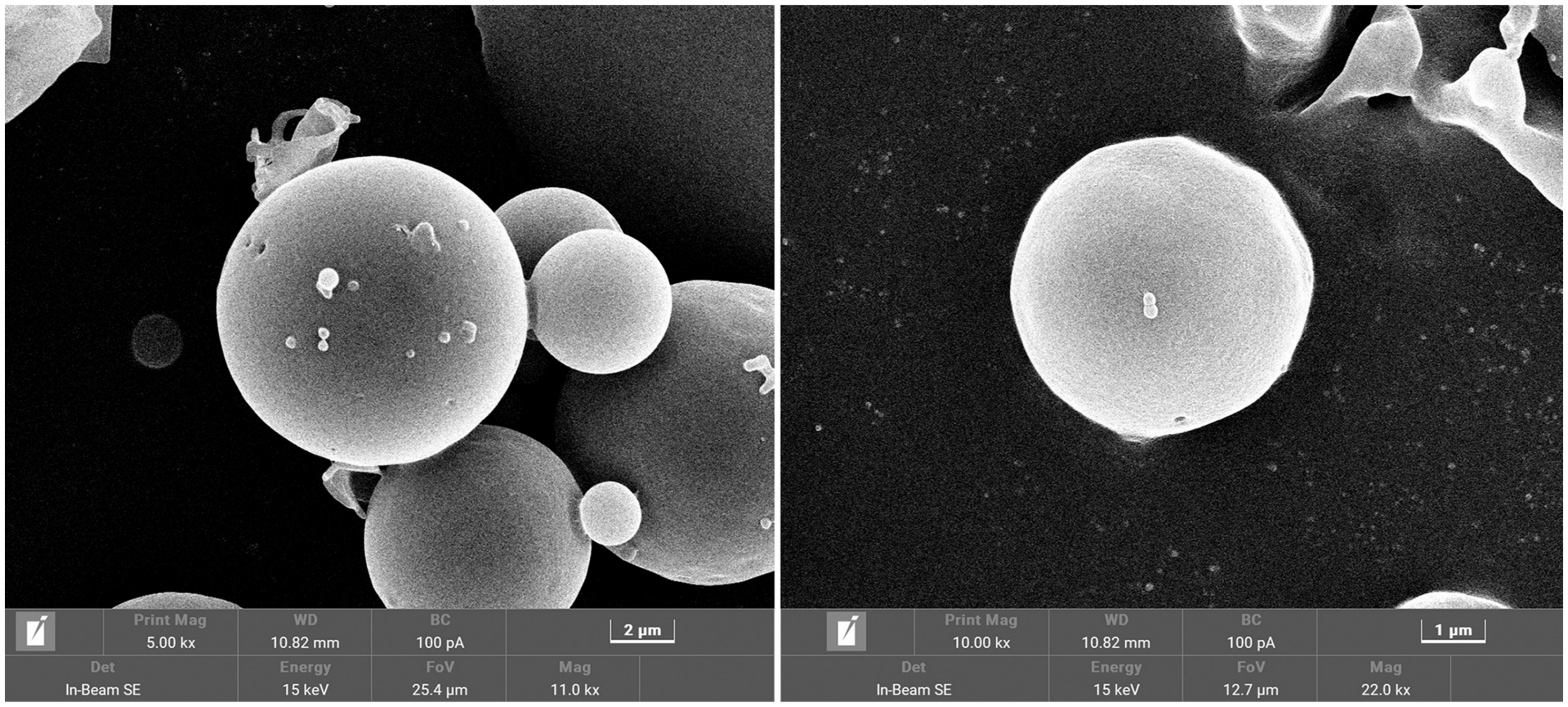
SEM of SRP-PLGA-MS.

**Figure 3:**
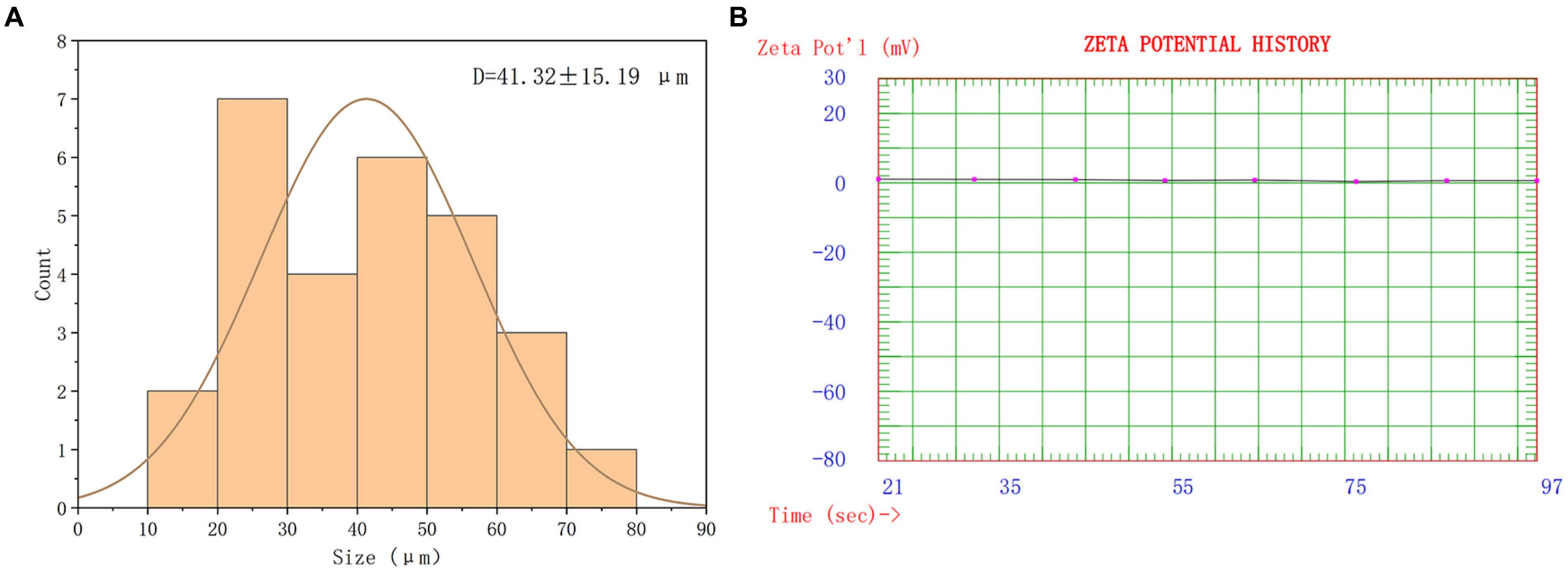
Size distribution of SRP-PLGA-MS **(A)**; Zeta-potential of SRP-PLGA-MS **(B)**.

The overall morphology of SRP-PLGA-MS appeared as regular spheres with a low surface porosity (Figure 2). The particle size of SRP-PLGA-MS was 41.32 ± 15.19 μm (Figure 3A), and the zeta potential was 0.47 ± 0.27 mA (Figure 3B).

### 3.2 *In vitro* drug release

**Figure 4:**
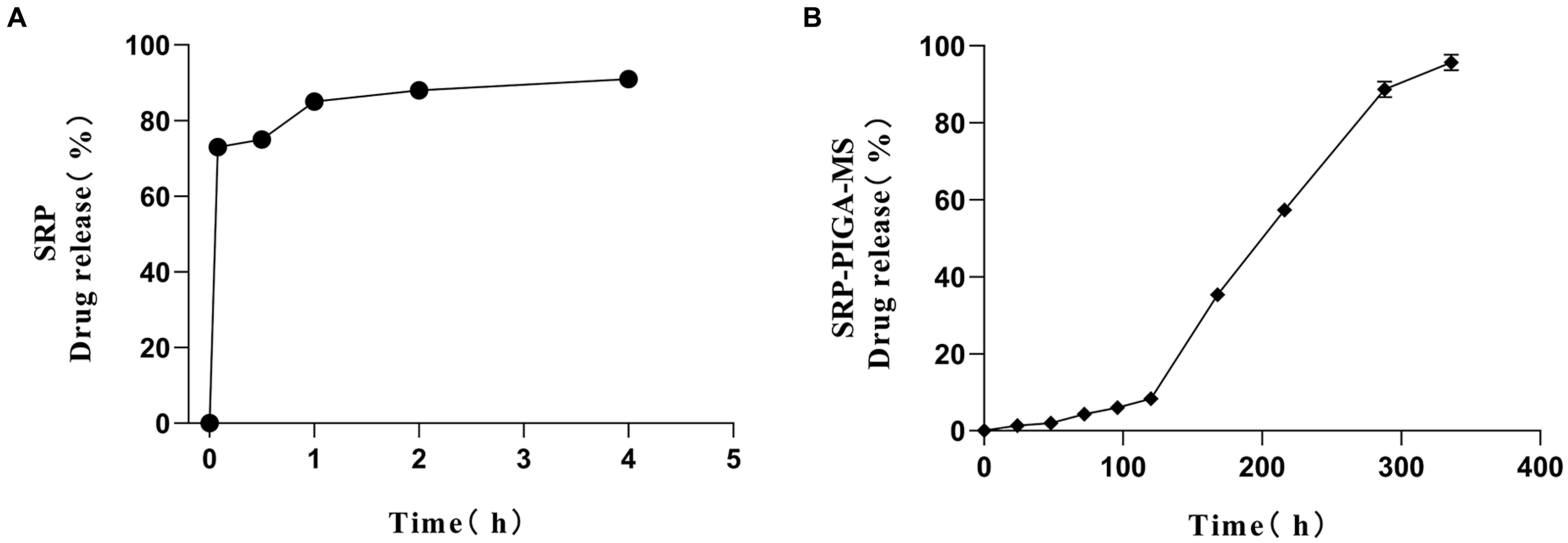
*In vitro* release rate of SRP **(A)**; *In vitro* drug release of SRP-PLGA-MS **(B)**.

The release kinetics of SRP-PLGA-MS in deionized water was compared with those of free SRP. As shown in Figure 4A, more than 90% of free SRP was released within 4 h of administration. Conversely, the cumulative release of SRP-PLGA-MS reached 96% within 14 d (Figure 4B). Within the period of 0–120 h, the amount of SRP-PLGA-MS released was approximately 20%. After 120 h, the amount released increased slowly, which may be attributed to the progressive degradation of the PLGA microspheres. Therefore, the entire process of drug release in SRP-PLGA-MS was slow and sustained. These results indicated that SRP-PLGA-MS had a slower sustained release than free SRP, which made it effective in overcoming the disadvantage of a short half-life.

### 3.3 Sustained and stable blood pressure reduction *in vivo* with SRP-PLGA-MS

**Figure 5:**
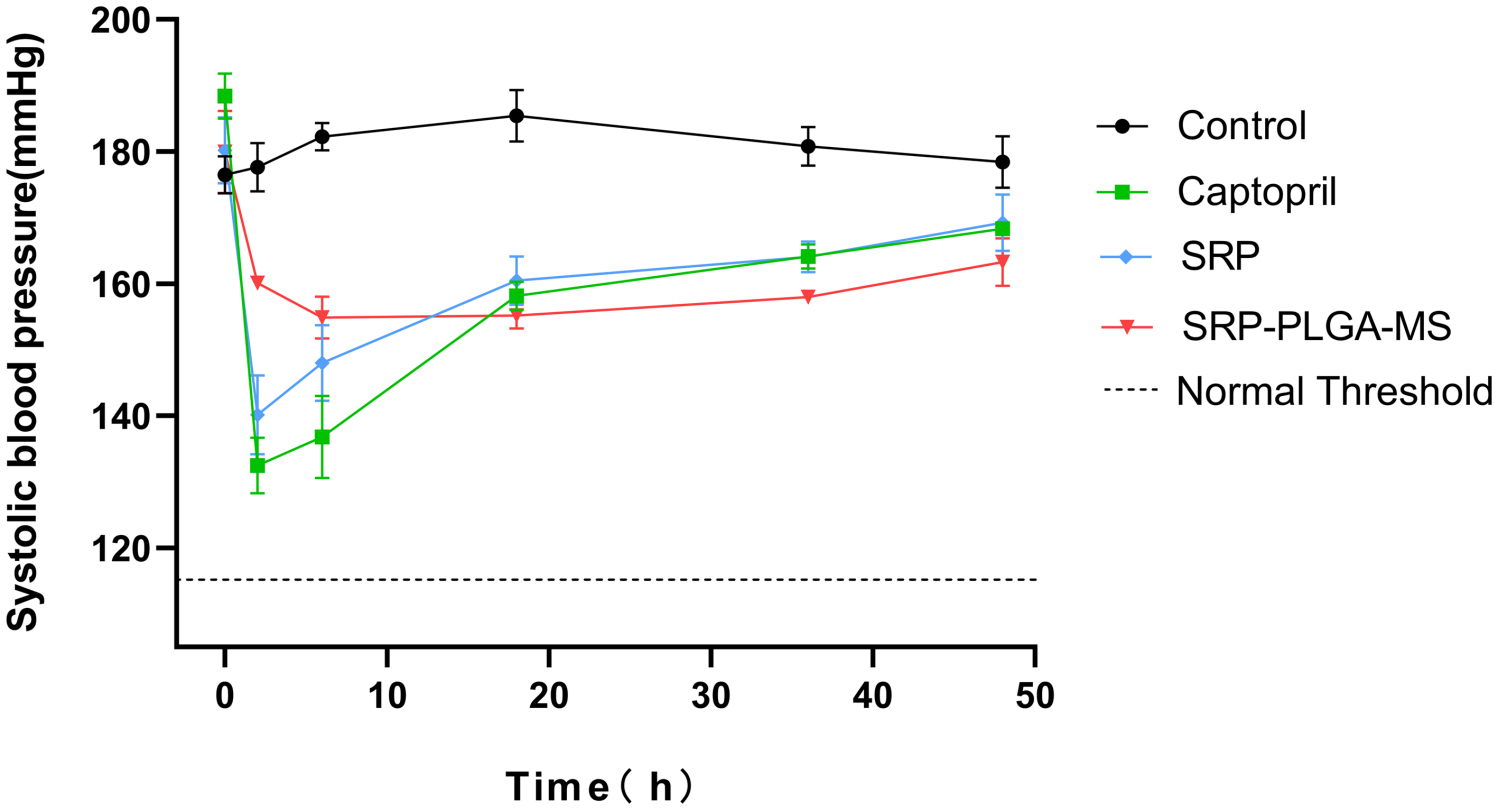
Systolic blood pressure changes in SHRs after a single oral administration (*n* = 4). The oral administration dose was 30 mg/kg body weight in Captopril group and SRP group, 10 mg SRP/kg in SRP-PLGA-MS group. SBP was measured at 0, 2, 6, 18, 36, and 48 h after administration. ^*^p < 0.05 in all groups compared to the control group.

*In vivo* antihypertensive effects were evaluated in SHR by monitoring the systolic blood pressure (SBP). As shown in Figure 5, after a single oral administration, SBP in control group was maintained at 180.17 ± 4.26 mmHg, with barely fluctuated in 48 h. SBP in Captopril group and SRP group was rapidly decreased within 2 h, their antihypertensive effect at 2 h was similar (Captopril: 132.50 ± 4.21 mmHg; SRP: 140.13 ± 5.96 mmHg), and then their blood pressure kept climbing. The SBP in SRP-PLGA-MS group slowly decreased to 154.85 ± 3.17 mmHg within 6 h and stabilized for 48 h. Obviously, the lowest point of SBP in SRP-PLGA-MS group was higher than that in SRP group, due to the SRP dosage in SRP-PLGA-MS group was about one third in SRP group. The results showed that after the concentrated release of Captopril and SRP, SBP fluctuated dramatically, which was unfavorable to blood pressure homeostasis. In contrast, the drug release of SRP-PLGA-MS was sustained *in vivo* and its antihypertensive effect was long-acting and smooth.

### 3.4 SRP-PLGA-MS improved vascular pathomorphology

**Figure 6:**
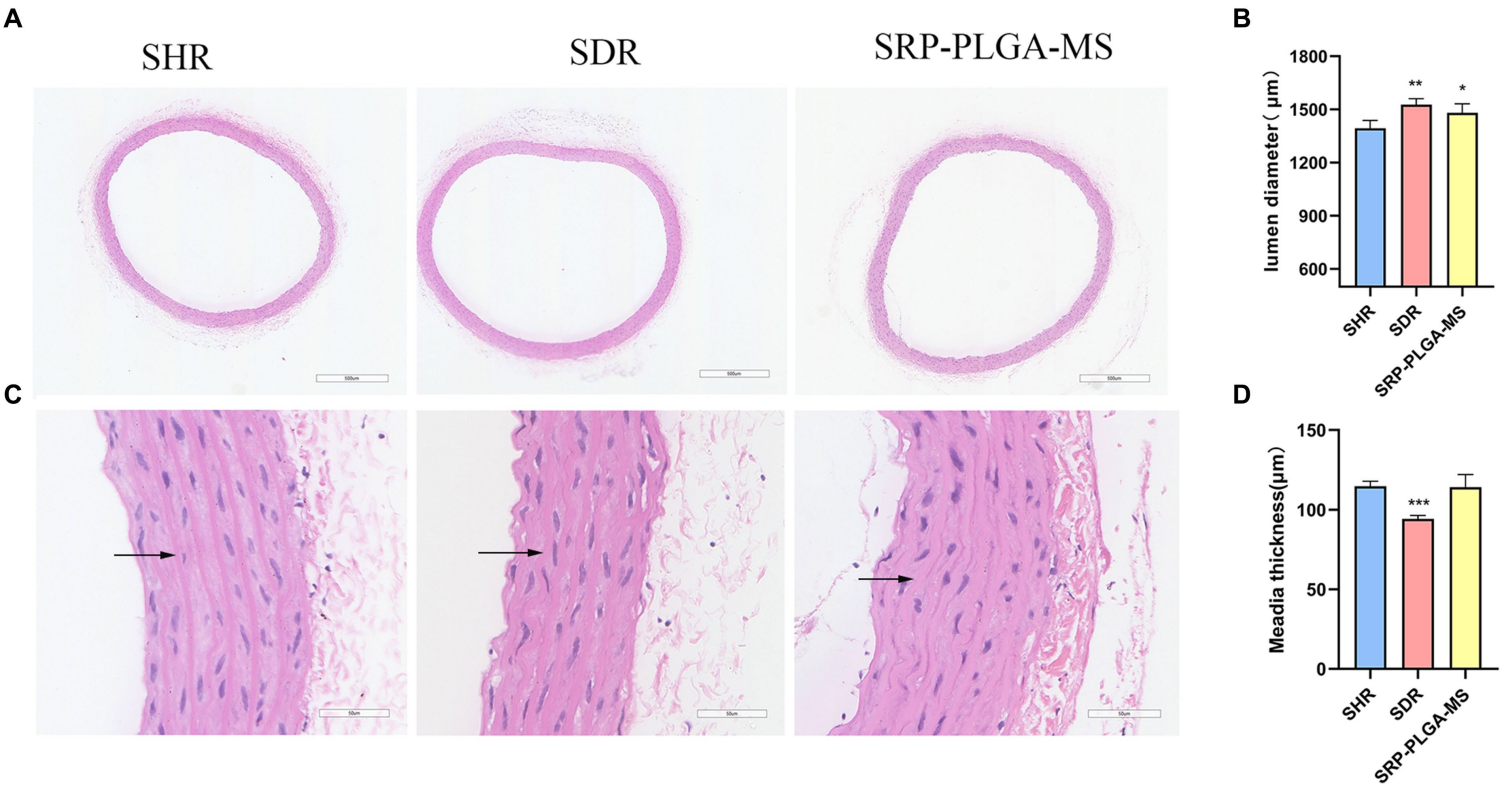
Immunohistochemical analysis and representative pathomorphology images of all groups (*n* = 4). **(A)** Representative photomicrograph of thoracic aorta sections stained with hematoxylin and eosin (H and E, ×4). **(C)** Representative photomicrograph of thoracic aorta sections stained with hematoxylin and eosin (H and E, ×40). Black arrows point to vascular elastic fibers. **(B)** The lumen diameter. **(D)** The media thickness. Values are expressed as the mean ± standard deviation. ^*^p < 0.05, ^**^p < 0.01, ^***^p < 0.001 compared with the model group (SHR).

The rat aortic vessel sections of all experimental groups were stained by hematoxylin and eosin (HE) to observe the vascular pathomorphology, their lumen diameter and media thickness were measured (Figure 6). In Figures 6A,B, the lumen diameter in SRP-PLGA-MS group was larger more than that in SHR group, and almost the same as that in SDR group. The results showed that the vascular lumen of SHR was narrow resulting in blood pressure high. After administration with SRP-PLGA-MS, the vascular lumen became enlarged to almost the same as the SDR. In Figures 6C,D, the media thickness in SRP-PLGA-MS group was almost the same as that in SHR group, yet larger more than that in SDR group. The results showed that the blood vessel wall of SHR was compensatory thickening due to long-term pressure of hypertension (26). As shown in Figure 6C black arrow, the vascular elastic fibers in SHR tensed to straight, while in SDR and SRP-PLGA-MS group, the vascular elastic fibers relaxed to bend. Our previous studies had confirmed that SRP was a good ACE inhibitor (IC_50_ = 0.046 mmol/L), in this research, the results indicated that ACE activity was inhibited *in vivo* leading to angiotensin decreased after administration with SRP-PLGA-MS, thus the vascular elastic fibers relaxed and the lumen of vessel expanded, thereby the blood pressure lowering (27). Other researcher found that ACE-inhibiting peptides altered the pathological morphology of the vessel wall when administered continuously for more than 1 week (11, 23). However, in this study, due to the short duration of administration, the media thickness of the vessel wall was not pathologically altered within 48 h of a single administration.

### 3.5 SRP-PLGA-MS effectively inhibits ACE and NADPH oxidase 4

**Figure 7:**
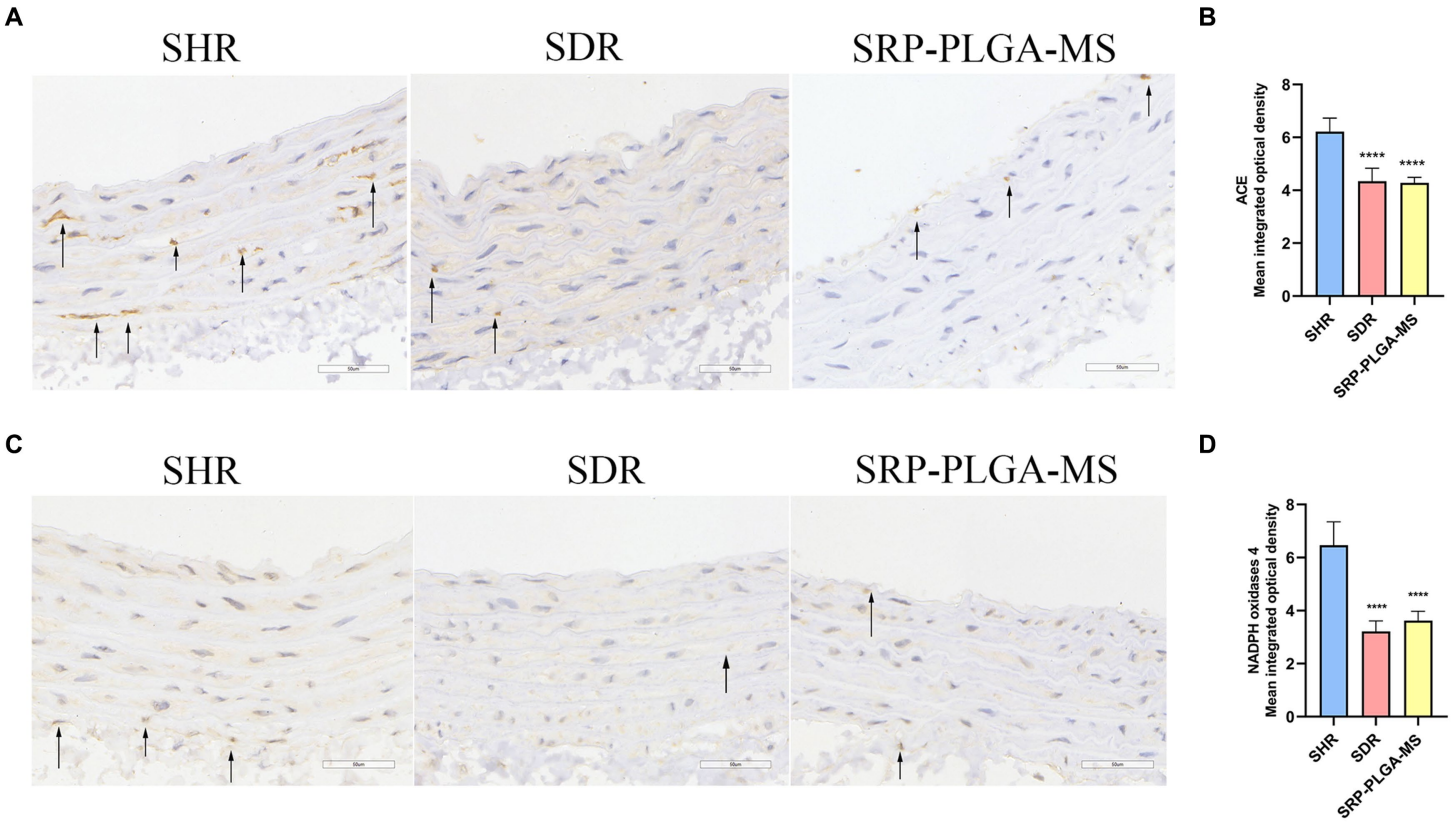
Effect of SRP-PLGA-MS on the ACE and NADPH oxidase 4 expression in thoracic aortic (*n* = 5). **(A)** Representative photomicrograph of the ACE expression in thoracic aortic by immunohistochemistry (×40); **(C)** Representative photomicrograph of the NADPH oxidase 4 expression in thoracic aortic by immunohistochemistry (×40); **(B)** Mean intergrated optical density of ACE; **(D)** Mean intergrated optical density of NADPH oxidase 4. ^###^*p* < 0.001 (), ^####^*p* < 0.0001 compared with the blank group normal control (SDR). ^***^p < 0.001, ^****^p < 0.0001 compared with the model group (SHR).

ACE was a key regulator of blood pressure, and NADPH oxidase 4 was an activator to induce ROS. Excess ROS caused oxidative stress and organ damaged. To further demonstrate the antihypertensive mechanism of SRP-PLGA-MS, the expression of ACE and NADPH oxidases 4 in the thoracic aorta was detected using IHC. In SHR group, ACE and NADPH oxidase 4 was highly expressed, while in SRP-PLGA-MS group, the expression of ACE and NADPH oxidases 4 significantly reduced, which were almost consistent with that of SDR group (Figures 7A–D). The results suggested that the formulation had the dual bioactivities of ACE inhibition and antioxidant.

### 3.6 Cytotoxicity assay of SRP

**Figure 8:**
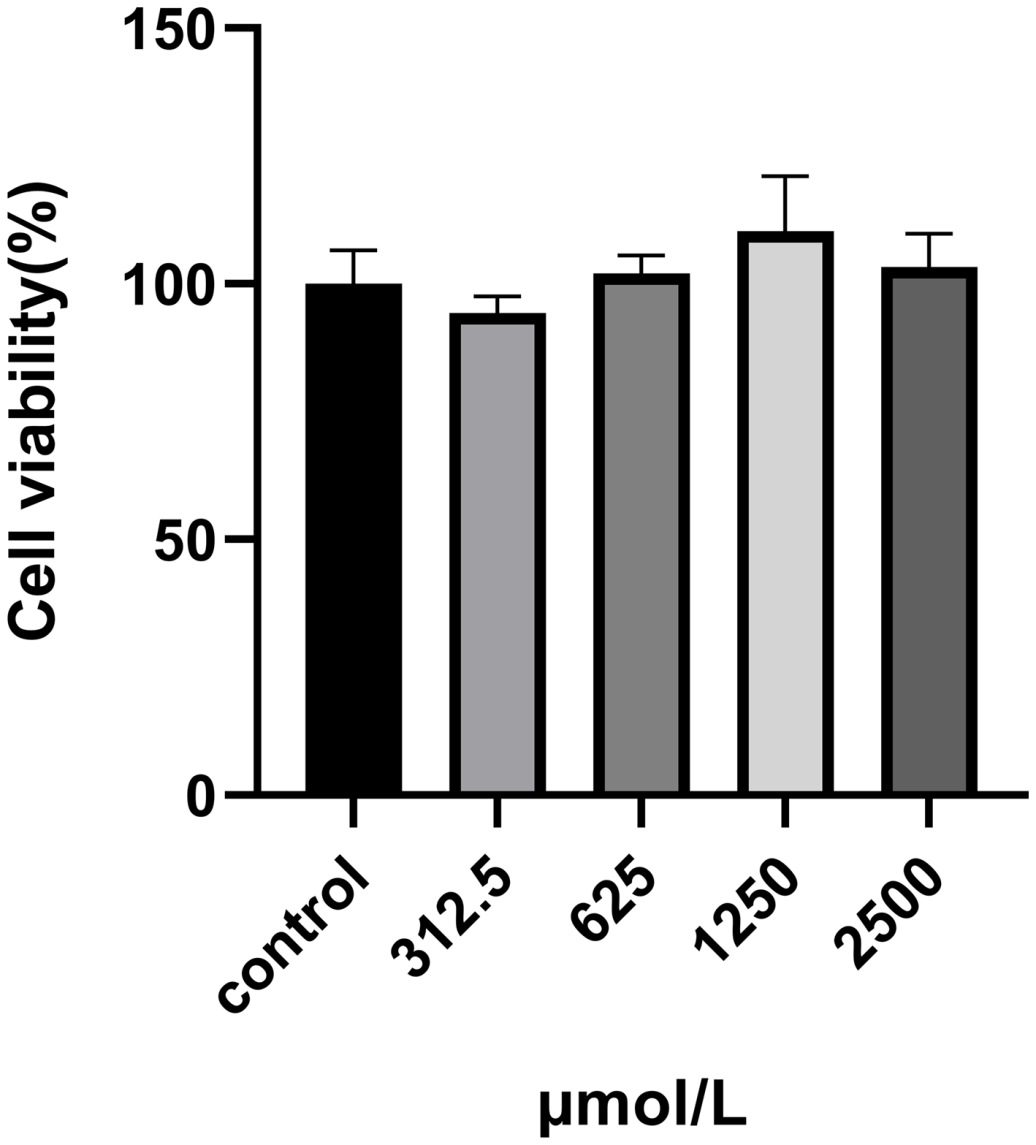
Effect of SRP on the viability of HUVECs.

The cytotoxicity of SRP in HUVECs was assayed using the MTT assay. This method was used to measure the activity of mitochondrial dehydrogenases in living cells. HUVECs were incubated with SRP for 24 h. All the concentrations showed similar trends. At high concentrations, the cell viability remained constant at approximately 80% (Figure 8). Therefore, it can be concluded that SRP had no toxic effect on HUVECs after 24 h in this study.

### 3.7 SRP effectively inhibits NO and ROS production in AngII-stimulated HUVECs

**Figure 9:**
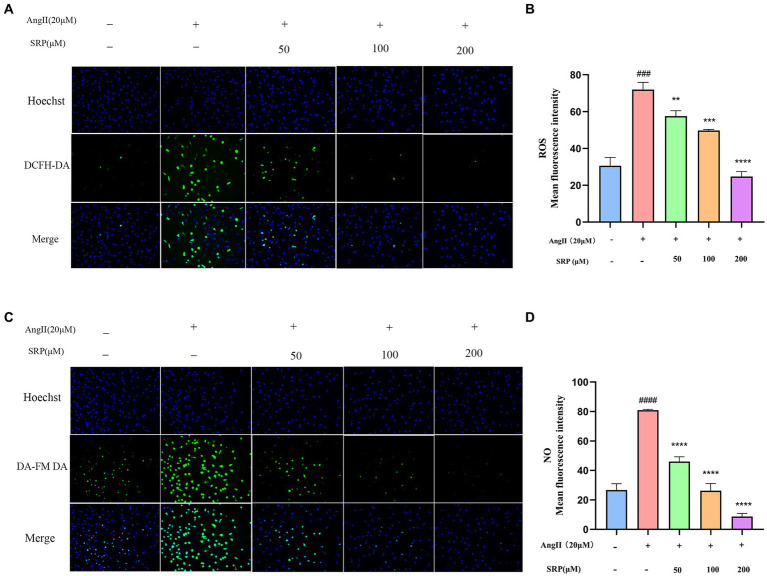
Intracellular total ROS and NO levels in HUVEC were detected by fluorescent probe DCFH-DA and DA-FM-DA (*n* = 3). Cells were co-incubated with AngII (20 μM), SRP (50, 100, 200 μM) for 24 h. Intracellular total ROS and NO fluorescence pictures were obtained by inverted fluorescence microscopy **(A,C)**. Mean fluorescence intensity of the fluorescence pictures **(B,D)**. ^###^*p* < 0.001 compared with the blank group (untreated cells), ^**^p < 0.01, ^***^p < 0.001, ^****^p < 0.0001, compared with the control group (AngII-treated cells).

In the fluorescence assay, HUVECs treated with AngII (20 μM) showed intense green fluorescence compared to untreated cells (Figures 9A,C). SRP treatment significantly reduced the mean optical density of ROS and NO from 71.97 to 24.75 and 80.89 to 8.72, respectively (Figures 9B,D). The results demonstrated that Ang II treatment produced more ROS and NO than in untreated HUVECs. The SRP treatment effectively inhibited ROS and NO production.

### 3.8 SRP regulates AngII-stimulated Nrf2 pathway expression in HUVECs

**Figure 10:**
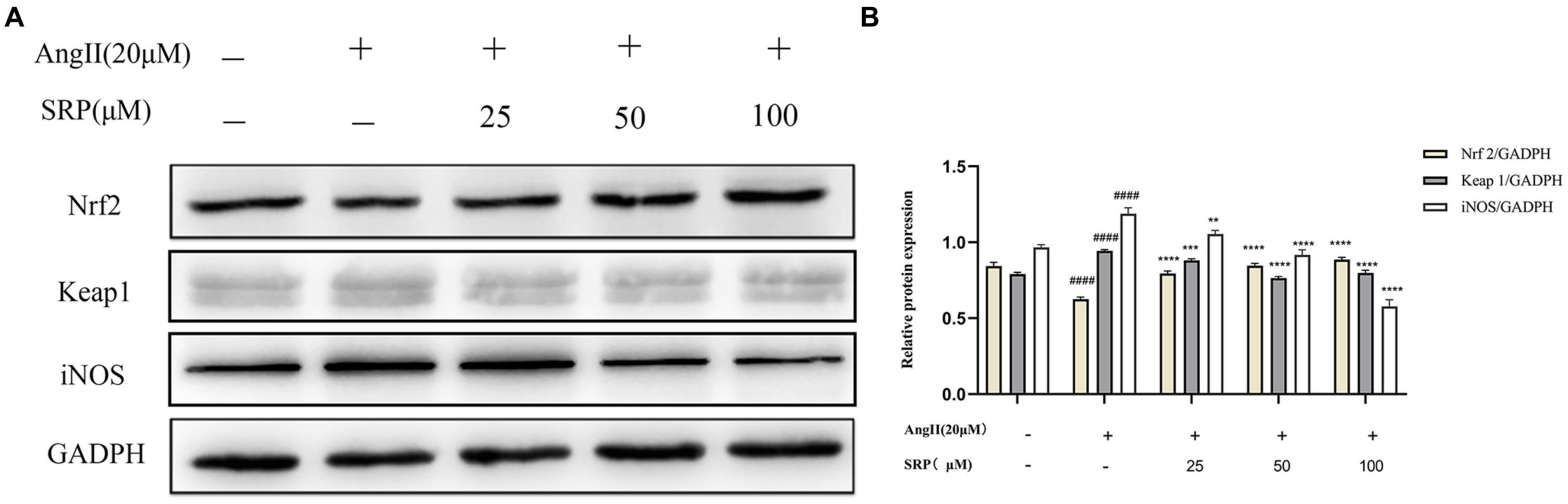
**(A)** Detection of the expression of iNOS, Nrf2, Keap1 by immunoblotting. **(B)** The ratios of Nrf2/GADPH, Keap1/GADPH, and iNOS/Tubulin were calculated (*n* = 3). Cells were co-incubated with AngII (20 μM), SRP (25, 50, 100 μM) for 24 h. ^####^*p* < 0.0001 compared with the blank group (untreated cells), ^**^p < 0.01, ^***^p < 0.001, ^****^p < 0.0001 compared with the control group (AngII-treated cells).

WB was used to examine the expression levels of a range of proteins (iNOS, Nrf2, and Keap1) in HUVECs. In AngII-induced HUVECs, SRP enhanced the expression of Nrf2, while reduced the expression of iNOS and Keap1. The results were showed in Figure 10, it indicated that SRP leaded to an increase of antioxidant components and decrease of NO, both of them were benefit to reducing oxidative stress.

## 4 Discussion

Poly-(lactic-co-glycolic acid) microspheres have been developed as controllable drug delivery carrier for active ingredients including some small molecules, peptides, and proteins, which generally have sustained release properties, and the duration of sustained release varies from a few days to several months (28). In this study, the formulation SRP-PLGA-MS was prepared and characterized (Figures 1–3), its morphology appeared as regular spheres with particle size approximately 41.32 ± 15.19 μm, and the zeta potential was 0.47 ± 0.27 mA. The drug release from PLGA-based formulations was extensively investigated, and the release mechanism was mainly defined as tri-phasic release (burst, lag, and erosion) (29). In this study, the duration of sustained release *in vitro* was more than 300 h (Figure 4). SRP release from the formulation showed a tri-phasic release profile, with an initial burst release (0–100 h), followed by a lag phase (100–300 h) and then an erosion release phase (beyond 300 h). Such tri-phasic release profiles are typical of PLGA microspheres (30).

Hypertension is a serious cardiovascular disease and vascular endothelial dysfunction is one of its important features (31–33). Endothelial dysfunction is attributed to chronic stimulation by vascular irritants, such as AngII, ONOO^−^ and other stimuli (34). ACE converts AngI to AngII, and then binds to AT1R, which produces a strong vasoconstrictor effect (6). Therefore, ACE inhibition is a potential pathway for antihypertension. An increasing number of natural ACE-inhibitory peptides have been studied (9, 10, 22). Our previous studies had confirmed that SRP was a good ACE inhibitor (IC_50_ = 0.046 mmol/L) (8, 35). In this study, the antihypertensive effect of SRP-PLGA-MS was verified in SHR animal experiments (Figure 5). The present study confirmed that vascular lumen was narrow due to vasoconstriction in spontaneously hypertensive rats, while SRP, similar to captopril, could reduce elastic fiber tightness to vasodilation (Figure 6), meanwhile, the expression of ACE in experimental animal was inhibited by administration with SRP-PLGA-MS formulation (Figures 7A,C). It indicated that the formulation possess sustained and smooth antihypertensive effect by inhibiting ACE activity.

Peroxynitrite (ONOO^−^) is a potent oxidizing and nitrifying agent that causes DNA damage and it was mainly generated by NO and superoxide radicals (O2^−^) (36–39). Thus, inhibition of NO and ROS could reduce the production of ONOO^−^ (36, 37, 40). The fluorescence data in this experiment confirmed that SRP decreased the production of NO and ROS within a safe concentration range (Figures 8, 9). The generation of NO and superoxide radicals (O2^−^) was regulated by iNOS and NADPH oxidase 4 (NOX4) respectively (37, 41). NOX4 was a human homolog of phagocyte NADPH oxidase and highly expressed in endothelial cells. ROS generation involved in many important signaling pathways related with NOX family (42). In this study, immunohistochemical analysis confirmed that SRP inhibited NOX4 expression (Figures 7C,D), and western blot analysis proved that SRP inhibited the expression of iNOS (Figure 10), which were benefit to antihypertension.

In addition, the imbalance between ROS and antioxidant enzymes caused oxidative stress, which was one of the etiologies of hypertension (43). Therefore, the activation of antioxidant enzymes to eliminate ROS was a conceivable therapeutic strategy (43, 44). Nrf2 is the primary target for ROS removal (45–49). It is a redox-sensitive transcription factor and a key therapeutic target for oxidative stress-related diseases. Under steady-state conditions, Nrf2 is mainly conserved in the cytoplasm by Keap1 (an inhibitor of Nrf2) in the cytoplasm. Under oxidative stress, Nrf2 dissociates from Keap1, and then translocate to the nucleus and combines with antioxidant-responsive elements, leading to the expression of antioxidant genes. Ultimately, a lot number of antioxidant components were produced to remove the excess ROS and alleviated the damage caused by oxidative stress (46, 50). In this study, Western blot analysis showed that SRP inhibited Keap1 expression and enhanced the dissociation of Nrf2 into the nucleus to express antioxidant genes (Figure 10), the results demonstrated that SRP was an effective agent in hypertension related with oxidative stress.

## 5 Conclusion

**Figure 11:**
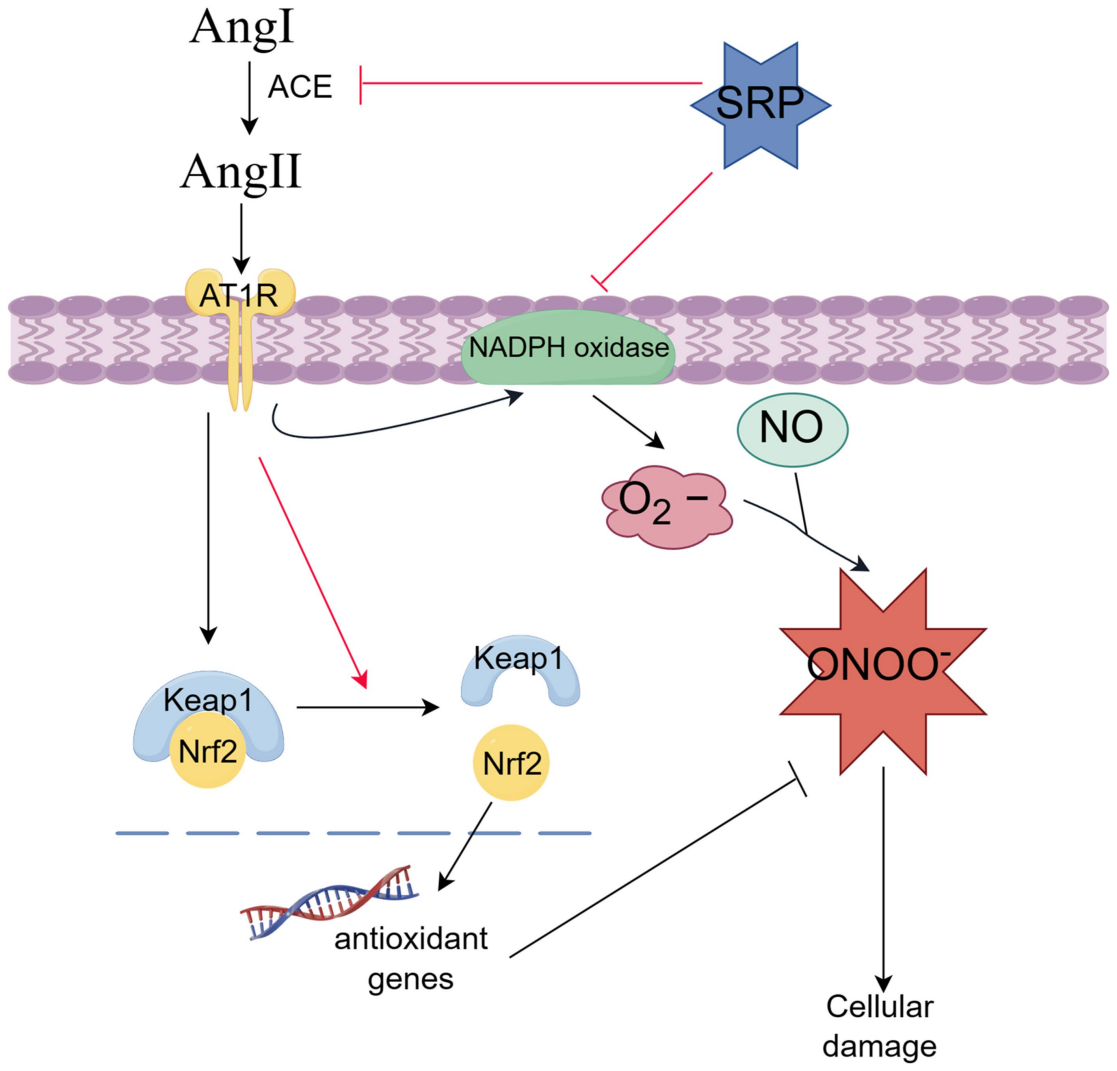
Antihypertension-related molecular mechanism of SRP (By Figdraw).

In this study, the sustained-release formulation (SRP-PLGA-MS) was prepared and characterized the physical and chemical properties. Its antihypertensive effect was verified in SHR animal experiments, and the antihypertensive mechanism was explored in AngII-induced HUVECs model. In conclusion, SRP-PLGA-MS was a potential antihypertensive preparation with sustained and smooth blood pressure lowing effect. The anti-hypertension mechanism of the agent was related with dual bioactivities of ACE inhibition and antioxidant involving NADPH oxidase and Keap1/Nrf2 signaling pathways (Figure 11).

## Data availability statement

The raw data supporting the conclusions of this article will be made available by the authors, without undue reservation.

## Ethics statement

Ethical approval was not required for the studies on humans in accordance with the local legislation and institutional requirements because only commercially available established cell lines were used. The animal study was approved by Guangzhou RAGE Biotechnology Co. The study was conducted in accordance with the local legislation and institutional requirements.

## Author contributions

MH: Writing – original draft, Writing – review & editing. TW: Investigation, Writing – review & editing. YW: Methodology, Writing – review & editing. QD: Data curation, Writing – review & editing. JC: Data curation, Writing – review & editing. HL: Project administration, Resources, Supervision, Writing – review & editing. YL: Writing – review & editing, Conceptualization, Data curation, Formal analysis, Funding acquisition, Investigation, Methodology, Project administration, Resources, Software, Supervision, Validation, Visualization, Writing – original draft. LL: Writing – review & editing, Supervision.
